# Preoperative neutrophil-to-lymphocyte ratio may contribute to the prediction of postoperative infectious complications in patients with acute appendicitis: a retrospective study

**DOI:** 10.1186/s12893-022-01529-8

**Published:** 2022-03-03

**Authors:** Mikito Mori, Kazuo Narushima, Atsushi Hirano, Yoshihiko Kano, Fumihiro Chiba, Yoshihiro Edamoto, Masahiro Yoshida

**Affiliations:** 1 Department of Surgery, Secomedic Hospital, 696-1 Toyotomi-cho, Funabashi, Chiba 274-0053 Japan; 2 Department of Hepato-Biliary-Pancreatic and Gastrointestinal Surgery, International University of Health and Welfare Ichikawa Hospital, 6-1-14 Kounodai, Ichikawa, Chiba 272-0827 Japan

**Keywords:** Acute appendicitis, Appendectomy, Neutrophils, Lymphocytes, Postoperative complications

## Abstract

**Background:**

Several studies have assessed various clinical variables to identify risk factors for postoperative complications in patients with acute appendicitis. However, few studies have focused on the relationships between systemic inflammatory variables and postoperative complications in patients with acute appendicitis. We investigated the relationships between postoperative complications and systemic inflammatory variables, and assessed the clinical utility of these variables as predictors of postoperative complications in patients with acute appendicitis.

**Methods:**

We retrospectively reviewed 181 patients who underwent immediate appendectomy for acute appendicitis. All postoperative complications were classified as infectious or noninfectious, and we evaluated the relationships between postoperative complications and clinical factors including the preoperative neutrophil-to-lymphocyte ratio and platelet-to-lymphocyte ratio.

**Results:**

In total, 28 patients (15.5%) had postoperative Clavien-Dindo grade II–IV complications; 17 patients (9.4%) and 11 patients (6.1%) were categorized as the infectious and noninfectious complication groups, respectively. The cutoff value of the preoperative neutrophil-to-lymphocyte ratio for all complications was 11.3, and multivariate analysis revealed that the preoperative neutrophil-to-lymphocyte ratio was an independent predictor of any postoperative complication (odds ratio: 4.223, 95% confidence interval: 1.335–13.352; *P* = 0.014). The cutoff value of the preoperative neutrophil-to-lymphocyte ratio for infectious complications was 11.4, and multivariate analysis revealed that the preoperative neutrophil-to-lymphocyte ratio was an independent predictor of infectious complications (odds ratio: 4.235, 95% confidence interval: 1.137–15.776; *P* = 0.031).

**Conclusions:**

In patients with acute appendicitis, the preoperative neutrophil-to-lymphocyte ratio may be a useful predictor of all postoperative complications, especially infectious complications.

## Background

Acute appendicitis is considered one of the most common diseases of the acute abdomen, with an estimated lifetime risk rate of 7–8% worldwide [1, 2]. Acute appendicitis can be clinically and epidemiologically classified as uncomplicated or complicated acute appendicitis, and 70–80% of all patients with appendicitis are diagnosed with uncomplicated acute appendicitis [3]. Although some randomized clinical trials have demonstrated that antibiotic therapy for acute appendicitis may be feasible as an alternative to immediate appendectomy [4–10], immediate appendectomy is still the standard treatment for acute appendicitis. Appendectomy is a cardinal surgical procedure with low mortality but a postoperative complication rate of 5–28% [5, 10–16]. Several studies have suggested that clinical variables such as age, operation timing, type of surgery, and type of acute appendicitis are risk factors for postoperative complications [11–16].

The neutrophil-to-lymphocyte ratio (NLR) and platelet-to-lymphocyte ratio (PLR) are well-known systemic inflammatory variables related to the prognosis of colorectal, gastric, and pancreatic cancer [17–19]. Some recent studies suggest that systemic inflammatory variables may be useful for the prediction of postoperative complications in patients with various cancers [20–22]. However, few studies have focused on the relationships between systemic inflammatory variables and postoperative complications in patients with acute appendicitis. Therefore, we investigated the relationships between postoperative complications and systemic inflammatory variables, and assessed the clinical utility of these variables as predictors of postoperative complications in patients with acute appendicitis.

## Methods

### Patients

The study protocol was approved by the Institutional Review Board of our institution. We retrospectively reviewed the electronic medical records of 181 patients with acute appendicitis who underwent appendectomy within 48 h after hospital admission between January 2010 and August 2021 at Secomedic Hospital, Japan. The patient inclusion criteria were age ≥ 15 years, emergency operation for acute appendicitis, and histologically confirmed diagnosis of acute appendicitis. We excluded patients who were pregnant, did not have histologically confirmed acute appendicitis, or underwent an interval appendectomy after initial antibiotic therapy. In this study, perforated appendicitis, gangrenous appendicitis, or appendicitis with a periappendiceal abscess was defined as complicated acute appendicitis; catarrhal or phlegmonous appendicitis was defined as uncomplicated acute appendicitis.

### Assessment of preoperative systemic inflammatory variables

A routine blood examination was performed at the time of hospitalization. We assessed the preoperative white blood cell count (pWBC), platelet count (pPLT), serum C-reactive protein level (pCRP), NLR (pNLR), and PLR (pPLR) as representative systemic inflammatory variables. As previously reported, the NLR and PLR were calculated by dividing the neutrophil and platelet count, respectively, by the lymphocyte count [17–19].

### Visual assessment of acute appendicitis on computed tomography images

In all patients, the maximum diameter of the appendix and the presence of fecalith, periappendiceal effusion, periappendiceal abscess, and ascites were evaluated on computed tomography (CT) images before surgery.

### Definitions of postoperative complications

All complications occurring within 30 days after surgery were defined as postoperative complications and were graded using the Clavien-Dindo classification (C-D) system [23]. A diagnosis of postoperative intra-abdominal abscess, cholangitis, or paralytic ileus was based on the patient’s symptoms, laboratory data, abdominal radiography, and CT findings. All postoperative complications were categorized as infectious or noninfectious.

### Statistical analysis

The relationships between postoperative complications and systemic inflammatory variables were evaluated by the area under the curves (AUCs) of the receiver operating characteristic (ROC) curves. The cutoff values of continuous variables (including systemic inflammatory variables) were set at the point on the ROC curve closest to the (0,1) point; these points corresponded to the optimal sensitivity and specificity yielding the minimal value for (1-sensitivity)^2^ + (1-specificity)^2^ [24]. The relationships between postoperative complications and type of appendectomy (open or laparoscopic) in patients with acute appendicitis were evaluated by Fisher’s exact test. Univariate analyses of postoperative complications were performed using logistic regression to determine whether there were significant associations between the clinical factors and postoperative complications. Multivariate analysis was performed using logistic regression to determine the statistical significance of the clinical factors identified by univariate analysis. *P* values < 0.05 indicated statistical significance. *P* values in multiple comparisons using Fisher’s exact test were corrected using a false discovery rate. All statistical analyses were performed using SPSS for Windows (version 26.0; IBM Corp., Armonk, NY, USA).

## Results

### Patient characteristics

The clinical characteristics of 181 patients (70 women, 111 men) with acute appendicitis who underwent immediate appendectomy are summarized in Table 1. Among 181 patients with acute appendicitis, 28 patients developed a postoperative complication classified as C-D grade II–IV.

**Table 1:** Characteristics of 181 patients with acute appendicitis

Variable	n = 181
Sex, male/female	111/70
Age, years	44 (15−92)
BMI, kg/m^2^	21.8 (15.6−33.6)
ASA-PS classification, IE/IIE/IIIE	72/103/6
pBT, ℃	37.2 (35.3−39.5)
pWBC, × 10^9^/L	13.5 (4.1−24.8)
pPLT, × 10^9^/L	231 (106−480)
pNTC, × 10^9^/L	11.2 (2.8−21.7)
pLPC, × 10^9^/L	1.2 (0.3−5.2)
pNLR	10.2 (1.8−38.2)
pPLR	202.0 (51.2−941.9)
pCRP, mg/L	47.6 (0.1−334.7)
Maximum diameter of the appendix, mm	9.8 (5.7−20.8)
Fecalith (+ /−)	95/86
Periappendiceal effusion (+ /−)	124/57
Periappendiceal abscess (+ /−)	13/168
Ascites (+ /−)	35/146
Time to operation, < 6/6−12/13−24/25−48 h	108/59/6/8
Type of appendectomy, open/laparoscopic	31/150
Operative time, minutes	50 (23−160)
Estimated blood loss, mL	1 (1−300)
Type of acute appendicitis, uncomplicated/complicated	93/88
Postoperative complications, C−D grade ≥ II (+ /−)	28 /153

Data are presented as n or median (range). BMI, body mass index; ASA-PS, American Society of Anesthesiologists physical status; pBT, preoperative body temperature; pWBC, preoperative white blood cell count; pPLT, preoperative platelet count; pNTC, preoperative neutrophil count; pLPC, preoperative lymphocyte count; pNLR, preoperative neutrophil-to-lymphocyte ratio; pPLR, preoperative platelet-to-lymphocyte ratio; pCRP, preoperative C-reactive protein level, C-D, Clavien-Dindo classification

### Relationships between postoperative complications and appendectomy type in patients with acute appendicitis

Among 28 patients with C-D grade ≥ II postoperative complications (15.5%), 17 patients (9.4%) were defined as the infectious complication group, including 11 patients with intra-abdominal abscess (6.1%), four with wound infection (2.2%), one with cholangitis (0.6%), and one with enteritis (0.6%). All 11 patients (6.1%) in the noninfectious complication group had paralytic ileus. In the ‘all complications’ group and the infectious complications group, the incidence of complications significantly differed between patients who underwent open versus laparoscopic appendectomy (*P* < 0.001). In the infectious complication group, patients who underwent open appendectomy had a higher incidence of intra-abdominal abscess (*P* < 0.023) and wound infection (*P* < 0.001) than those who underwent laparoscopic appendectomy. In the noninfectious complication group, the incidence of paralytic ileus significantly differed between patients who underwent open versus laparoscopic appendectomy (*P* = 0.023) (Table 2).

**Table 2:** Relationships between postoperative complications and appendectomy type in patients with acute appendicitis

C−D grade ≥ II	Total n = 181	Open appendectomy n = 31	Laparoscopic appendectomy n = 150	*p* value^b^
All complications	28 (15.5%)	14 (45.2%)	14 (9.3%)	< 0.001^a^
Infectious complications	17 ( 9.4%)	9 (29.0%)	8 (5.3%)	< 0.001^a^
Intra-abdominal abscess	11 ( 6.1%)	5 (16.1%)	6 (4.0%)	0.023^a^
Wound infection	4 (2.2%)	4 (12.9%)	0 (0.0%)	< 0.001^a^
Cholangitis	1 (0.6%)	0 (0.0%)	1 (0.7%)	1.000
Enteritis	1 (0.6%)	0 (0.0%)	1 (0.7%)	1.000
Noninfectious complications	11 (6.1%)	5 (16.1%)	6 (4.0%)	0.023^a^
Paralytic ileus	11 (6.1%)	5 (16.1%)	6 (4.0%)	0.023^a^

Data are presented as n (%). C-D, Clavien-Dindo classification

^a^Statistically significant after false discovery rate correction. ^b^Fisher’s exact test

### Identification of useful predictors of all postoperative complications in patients with acute appendicitis

**Fig. 1:**
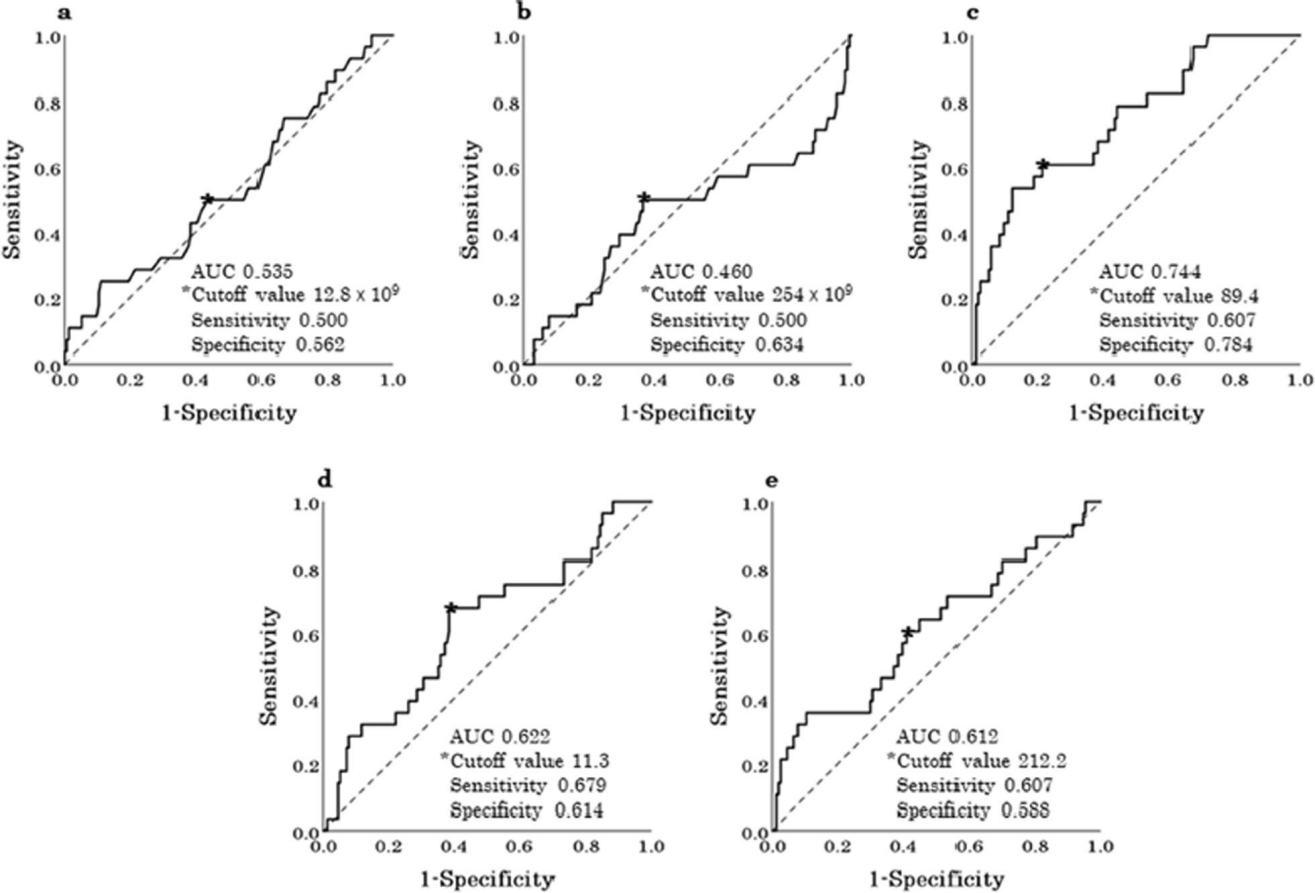
Determination of the cutoff values of the systemic inflammatory variables for all postoperative complications using receiver operating characteristic curves. **a** pWBC, **b** pPLT, **c** pCRP, **d** pNLR, **e** pPLR

Based on the ROC curves, the cutoff values of the pWBC, pPLT, pCRP, pNLR, and pPLR for all complications were set at 12.8 × 10^9^/L, 254 × 10^9^/L, 89.4 mg/L, 11.3, and 212.2, respectively (Fig. 1). Of the 20 clinical factors assessed using univariate analysis, the variables significantly associated with all postoperative complications were age (odds ratio (OR), 4.856; 95% confidence interval (CI) 2.003–11.772; *P* < 0.001), body mass index (BMI) (OR, 2.590; 95% CI 1.141–5.879; *P* = 0.023), American Society of Anesthesiologists physical status classification (OR, 2.486; 95% CI 1.137–5.437; *P* = 0.023), preoperative body temperature (OR, 3.043; 95% CI 1.263–7.334; *P* = 0.013), pNLR (OR, 3.363; 95% CI 1.427–7.927; *P* = 0.006), pCRP (OR, 5.409; 95% CI 2.315–12.640; *P* < 0.001), presence of periappendiceal effusion (OR, 4.545; 95% CI, 1.312–15.748; *P* = 0.017), presence of periappendiceal abscess (OR, 3.940; 95% CI 1.186–13.093; *P* = 0.025), presence of ascites (OR, 2.844; 95% CI 1.175–6.884; *P* = 0.020), type of appendectomy (OR, 0.125; 95% CI 0.051–0.306; *P* < 0.001), operative time (OR, 8.195; 95% CI 3.242–20.713; *P* < 0.001), estimated blood loss (OR, 6.202; 95% CI 2.622–14.672; *P* < 0.001), and complicated acute appendicitis (OR, 8.344; 95% CI 2.760–25.221; *P* < 0.001). Multivariate analysis of the significant factors identified using univariate analysis revealed that the independent risk factors for all postoperative complications were BMI (OR, 3.623; 95% CI 1.116–11.757; *P* = 0.032), pNLR (OR, 4.223; 95% CI 1.335–13.352; *P* = 0.014), operative time (OR, 4.850; 95% CI 1.410–16.682; *P* = 0.012), and complicated acute appendicitis (OR, 4.590; 95% CI 1.004–20.972; *P* = 0.049) (Table 3).

**Table 3:** Relationships between all complications and clinical factors in patients with acute appendicitis

Variable	All complications		Univariate analysis	*p* value^b^	Multivariate analysis	*p* value^b^
	+	−	OR (95% CI)		OR (95% CI)	
Sex, male/female	18/10	93/60	0.861 (0.372−1.991)	0.727		
Age, < 49/ ≥ 49 years	8/20	101/52	4.856 (2.003−11.772)	< 0.001^a^	3.672 (0.743−18.134)	0.110
BMI, < 23.1/ ≥ 23.1 kg/m^2^	12/16	101/52	2.590 (1.141−5.879)	0.023^a^	3.623 (1.116−11.757)	0.032^a^
ASA-PS classification, IE/IIE/IIIE	5/22/1	67/81/5	2.486 (1.137−5.437)	0.023^a^	0.387 (0.080−1.864)	0.236
pBT, < 37.3/ ≥ 37.3 °C	8/20	84/69	3.043 (1.263−7.334)	0.013^a^	2.351 (0.732−7.545)	0.151
pWBC, < 12.8/ ≥ 12.8 × 10^9^/L	14/14	67/86	0.779 (0.348−1.746)	0.544		
pPLT, < 254/ ≥ 254 × 10^9^/L	14/14	96/57	1.684 (0.749−3.786)	0.207		
pNLR, < 11.3/ ≥ 11.3	9/19	94/59	3.363 (1.427−7.927)	0.006^a^	4.223 (1.335−13.352)	0.014^a^
pPLR, < 212.2/ ≥ 212.2	11/17	89/64	2.149 (0.943−4.897)	0.069		
pCRP, < 89.4/ ≥ 89.4 mg/L	11/17	119/34	5.409 (2.315−12.640)	< 0.001^a^	1.108 (0.346−3.551)	0.863
Maximum diameter of the appendix, < 10.0/ ≥ 10.0 mm	10/18	82/71	2.079 (0.901−4.795)	0.086		
Fecalith (+ / −)	18/10	77/76	1.777 (0.770−4.097)	0.178		
Periappendiceal effusion (+ /−)	25/3	99/54	4.545 (1.312−15.748)	0.017^a^	0.345 (0.053−2.252)	0.266
Periappendiceal abscess (+ /−)	5/23	8/145	3.940 (1.186−13.093)	0.025^a^	1.453 (0.264−7.989)	0.667
Ascites (+ / −)	10/18	25/128	2.844 (1.175−6.884)	0.020^a^	2.121 (0.607−7.411)	0.239
Time to operation, < 6/6−12/13−24/25−48 h	17/10/0/1	91/49/6/7	0.877 (0.501−1.538)	0.648		
Type of appendectomy, open/laparoscopic	14/14	17/136	0.125 (0.051−0.306)	< 0.001^a^	0.691 (0.055−8.744)	0.776
Operative time, < 60/ ≥ 60 min	7/21	112/41	8.195 (3.242−20.713)	< 0.001^a^	4.850 (1.410−16.682)	0.012^a^
Estimated blood loss, < 3/ ≥ 3 mL	13/15	129/24	6.202 (2.622−14.672)	< 0.001^a^	1.590 (0.126−20.000)	0.720
Type of acute appendicitis, uncomplicated/complicated	4/24	89/64	8.344 (2.760−25.221)	< 0.001^a^	4.590 (1.004−20.972)	0.049^a^

Data are presented as n

BMI, body mass index; ASA-PS, American Society of Anesthesiologists physical status; pBT, preoperative body temperature; pWBC, preoperative white blood cell count; pPLT, preoperative platelet count; pNLR, preoperative neutrophil-to-lymphocyte ratio; pPLR, preoperative platelet-to-lymphocyte ratio; pCRP, preoperative C-reactive protein level; OR, odds ratio; CI, confidence interval

^a^Statistically significant. ^b^Logistic regression

### Identification of useful predictors of infectious complications in patients with acute appendicitis

**Fig. 2:**
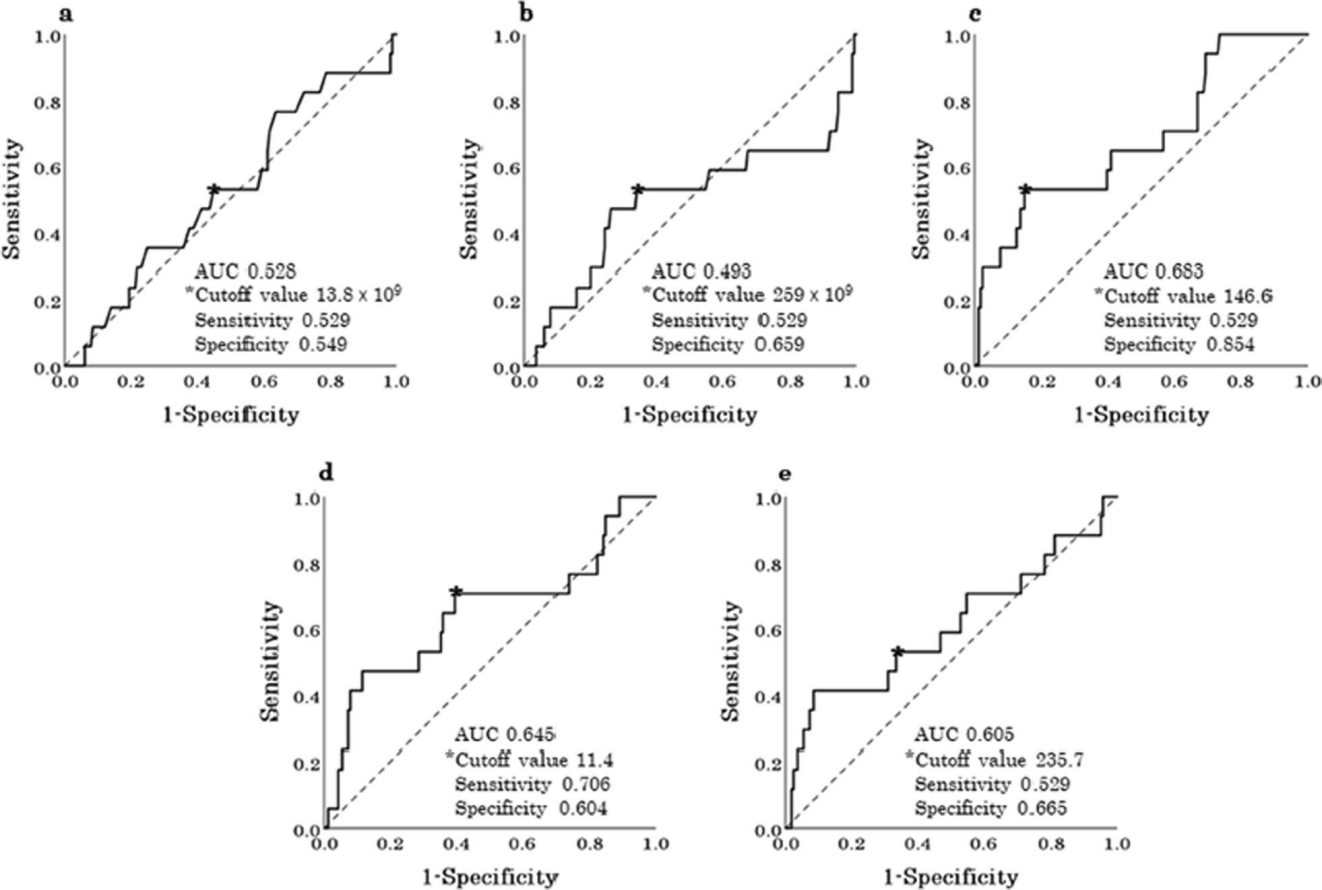
Determination of the cutoff values of the systemic inflammatory variables for infectious complications using receiver operating characteristic curves. **a** pWBC, **b** pPLT, **c** pCRP, **d** pNLR, **e** pPLR

The cutoff values of the pWBC, pPLT, pCRP, pNLR, and pPLR for the infectious complication group were set at 13.8 × 10^9^/L, 259 × 10^9^/L, 146.6 mg/L, 11.4, and 235.7, respectively (Fig. 2). Univariate analysis showed that the variables significantly associated with infectious complications were age (OR, 4.160; 95% CI 1.398–12.380; *P* = 0.010), BMI (OR, 4.629; 95% CI 1.553–13.795; *P* = 0.006), pNLR (OR, 3.655; 95% CI 1.230–10.863; *P* = 0.020), pCRP (OR, 6.255; 95% CI 2.204–17.754; *P* = 0.001), type of appendectomy (OR, 0.138; 95% CI 0.048–0.395; *P* < 0.001), operative time (OR, 7.628; 95% CI 2.369–24.563; *P* = 0.001), estimated blood loss (OR, 6.650; 95% CI 2.337–18.926; *P* < 0.001), and complicated acute appendicitis (OR, 5.676; 95% CI 1.571–20.502; *P* = 0.008). Multivariate analysis of the significant factors identified using univariate analysis revealed that the independent risk factors for infectious complications were BMI (OR, 4.471; 95% CI 1.275–15.676; *P* = 0.019) and pNLR (OR, 4.235; 95% CI 1.137–15.776; *P* = 0.031) (Table 4).

**Table 4:** Relationships between infectious complications and clinical factors in patients with acute appendicitis

Variable	All complications		Univariate analysis	*p* value^b^	Multivariate analysis	*p* value^b^
	+	−	OR (95% CI)		OR (95% CI)	
Sex, male/female	7/10	63/101	1.122 (0.406−3.099)	0.824		
Age, < 49/ ≥ 49 years	5/12	104/60	4.160 (1.398−12.380)	0.010^a^	1.210 (0.315−4.648)	0.782
BMI, < 23.1/ ≥ 23.1 kg/m^2^	5/12	108/56	4.629 (1.553−13.795)	0.006^a^	4.471 (1.275−15.676)	0.019^a^
ASA-PS classification, IE/IIE/IIIE	3/14/0	69/89/6	2.027 (0.793−5.185)	0.140		
pBT, < 37.3/ ≥ 37.3 °C	6/11	86/78	2.021 (0.714−5.724)	0.185		
pWBC, < 13.8/ ≥ 13.8 × 10^9^/L	8/9	86/78	1.240 (0.456−3.373)	0.673		
pPLT, < 259/ ≥ 259 × 10^9^/L	8/9	107/57	2.112 (0.773−5.770)	0.145		
pNLR, < 11.4/ ≥ 11.4	5/12	99/65	3.655 (1.230−10.863)	0.020^a^	4.235 (1.137−15.776)	0.031^a^
pPLR, < 235.7/ ≥ 235.7	8/9	108/56	2.170 (0.794−5.931)	0.131		
pCRP, < 146.6/ ≥ 146.6 mg/L	8/9	139/25	6.255 (2.204−17.754)	0.001^a^	2.160 (0.532−8.777)	0.282
Maximum diameter of the appendix, < 9.4/ ≥ 9.4 mm	3/14	68/96	3.306 (0.914−11.949)	0.068		
Fecalith (+ /−)	8/9	87/77	0.787 (0.289−2.140)	0.638		
Periappendiceal effusion (+ /−)	15/2	109/55	3.784 (0.836−17.141)	0.084		
Periappendiceal abscess (+ /−)	2/15	11/153	1.855 (0.375−9.160)	0.449		
Ascites (+ /−)	5/12	30/134	1.861 (0.610−5.680)	0.275		
Time to operation, < 6/6−12/13−24/25−48 h	11/6/0/0	97/53/6/8	0.669 (0.297−1.510)	0.334		
Type of appendectomy, open/laparoscopic	9/8	22/142	0.138 (0.048−0.395)	< 0.001^a^	1.588 (0.127−19.912)	0.720
Operative time, < 60/ ≥ 60 min	4/13	115/49	7.628 (2.369−24.563)	0.001^a^	3.704 (0.854−16.066)	0.080
Estimated blood loss, < 3/ ≥ 3 mL	7/10	135/29	6.650 (2.337−18.926)	< 0.001^a^	3.924 (0.324−47.571)	0.283
Type of acute appendicitis, uncomplicated/complicated	3/14	90/74	5.676 (1.571−20.502)	0.008^a^	1.583 (0.331−7.570)	0.565

Data are presented as n

BMI, body mass index; ASA-PS, American Society of Anesthesiologists physical status; pBT, preoperative body temperature; pWBC, preoperative white blood cell count; pPLT, preoperative platelet count; pNLR, preoperative neutrophil-to-lymphocyte ratio; pPLR, preoperative platelet-to-lymphocyte ratio; pCRP, preoperative C-reactive protein level; OR, odds ratio; CI, confidence interval

^a^Statistically significant. ^b^Logistic regression

### Identification of useful predictors of noninfectious complications in patients with acute appendicitis

**Fig. 3:**
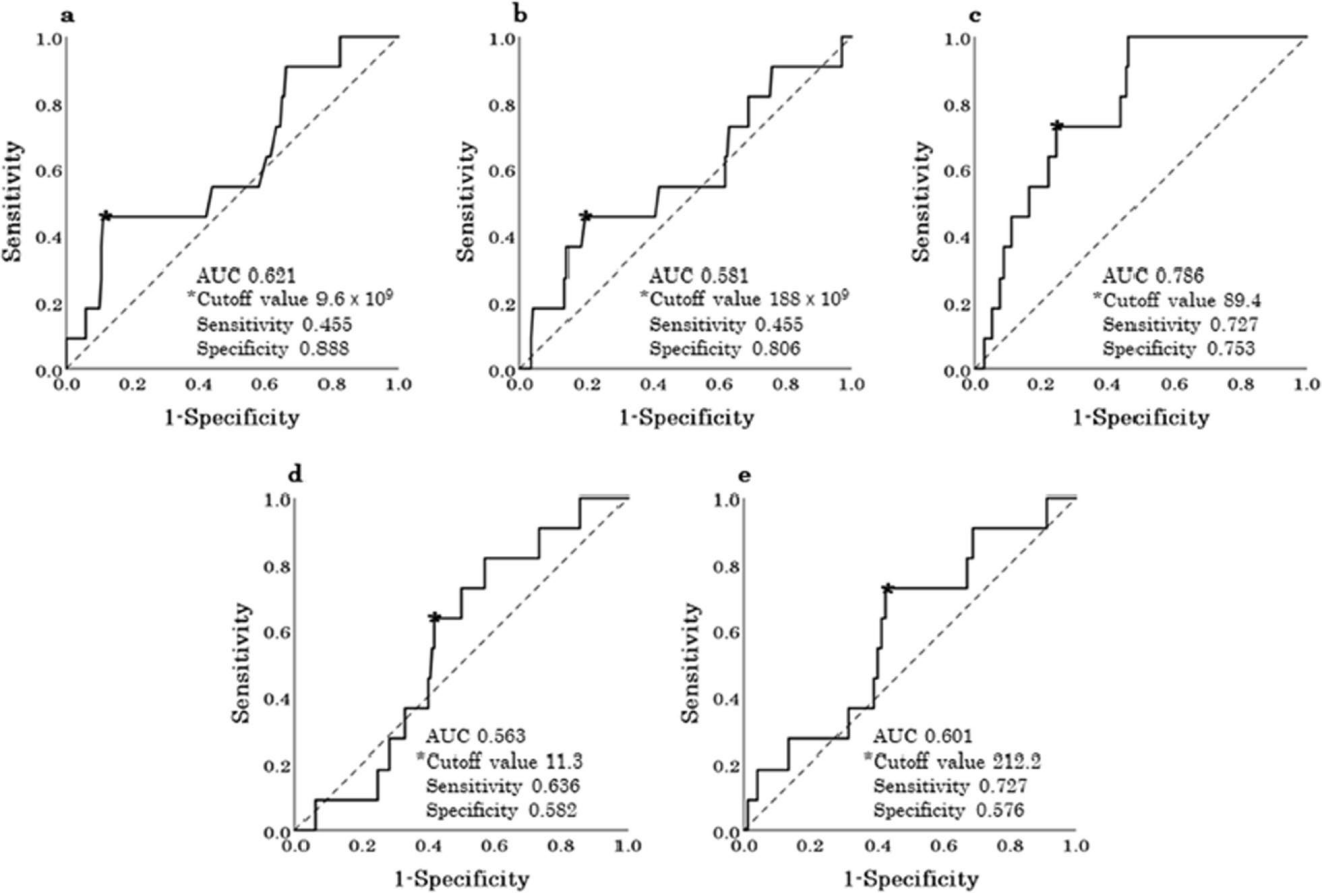
Determination of the cutoff values of the systemic inflammatory variables for noninfectious complications using receiver operating characteristic curves. **a** pWBC, **b** pPLT, **c** pCRP, **d** pNLR, **e** pPLR

The cutoff values of the pWBC, pPLT, pCRP, pNLR, and pPLR for the noninfectious complication group were set at 9.6 × 10^9^/L, 188 × 10^9^/L, 89.4 mg/L, 11.3, and 212.2, respectively (Fig. 3). Univariate analysis showed that the variables significantly associated with noninfectious complications were age (OR, 5.149; 95% CI 1.316–20.149; *P* = 0.019), preoperative body temperature (OR, 5.062; 95% CI 1.062–24.128; *P* = 0.042), pWBC (OR, 0.151; 95% CI 0.042–0.543; *P* = 0.004), pCRP (OR, 7.876; 95% CI 1.999–31.033; *P* = 0.003), presence of fecalith (OR, 10.000; 95% CI 1.252–79.845; *P* = 0.030), presence of periappendiceal abscess (OR, 6.000; 95% CI 1.376–26.166; *P* = 0.017), presence of ascites (OR, 3.889; 95% CI 1.114–13.582; *P* = 0.033), type of appendectomy (OR, 0.217; 95% CI 0.062–0.762; *P* = 0.017), operative time (OR, 7.265; 95% CI 2.008–26.285; *P* = 0.003), and complicated acute appendicitis (OR, 11.795; 95% CI 1.477–94.190; *P* = 0.020). Multivariate analysis of the significant factors identified using univariate analysis revealed that the only independent risk factor for noninfectious complications was the pWBC (OR, 0.125; 95% CI 0.021–0.729; *P* = 0.021) (Table 5).

**Table 5:** Relationships between noninfectious complications and clinical factors in patients with acute appendicitis

Variable	All complications		Univariate analysis	*p* value^b^	Multivariate analysis	*p* value^b^
	+	−	OR (95% CI)		OR (95% CI)	
Sex, male/female	8/3	103/67	0.576 (0.148−2.251)	0.428		
Age, < 51/ ≥ 51 years	3/8	112/58	5.149 (1.316−20.149)	0.019^a^	2.157 (0.343−13.563)	0.413
BMI, < 21.4/ ≥ 21.4 kg/m^2^	6/5	75/95	0.658 (0.193−2.239)	0.503		
ASA-PS classification, IE/IIE/IIIE	2/8/1	70/95/5	2.732 (0.848−8.803)	0.092		
pBT, < 37.3/ ≥ 37.3 °C	2/9	90/80	5.062 (1.062−24.128)	0.042^a^	4.100 (0.646−26.016)	0.134
pWBC, < 9.6/ ≥ 9.6 × 10^9^/L	5/6	19/151	0.151 (0.042−0.543)	0.004^a^	0.125 (0.021−0.729)	0.021^a^
pPLT, < 188/ ≥ 188 × 10^9^/L	5/6	33/137	0.289 (0.083−1.005)	0.051		
pNLR, < 11.3/ ≥ 11.3	4/7	99/71	2.440 (0.688−8.652)	0.167		
pPLR, < 212.2/ ≥ 212.2	3/8	97/73	3.543 (0.908−13.822)	0.069		
pCRP, < 89.4/ ≥ 89.4 mg/L	3/8	127/43	7.876 (1.999−31.033)	0.003^a^	3.065 (0.476−19.740)	0.239
Maximum diameter of the appendix, < 10.0/ ≥ 10.0 mm	3/8	89/81	2.930 (0.752−11.423)	0.121		
Fecalith (+ /−)	10/1	85/85	10.000 (1.252−79.845)	0.030^a^	7.950 (0.712−88.792)	0.092
Periappendiceal effusion (+ /−)	10/1	114/56	4.912 (0.613−39.334)	0.134		
Periappendiceal abscess (+ /−)	3/8	10/160	6.000 (1.376−26.166)	0.017^a^	2.617 (0.335−20.441)	0.359
Ascites (+ /−)	5/6	30/140	3.889 (1.114−13.582)	0.033^a^	2.013 (0.397−10.200)	0.398
Time to operation, < 6/6−12/13−24/25−48 h	6/4/0/1	102/55/6/7	1.203 (0.582−2.487)	0.618		
Type of appendectomy, open/laparoscopic	5/6	26/144	0.217 (0.062−0.762)	0.017^a^	0.932 (0.140−6.205)	0.942
Operative time, < 69/ ≥ 69 min	4/7	137/33	7.265 (2.008−26.285)	0.003^a^	1.884 (0.301−11.299)	0.508
Estimated blood loss, < 2/ ≥ 2 mL	6/5	135/35	3.214 (0.927−11.148)	0.066		
Type of acute appendicitis, uncomplicated/complicated	1/10	92/78	11.795 (1.477−94.190)	0.020^a^	1.877 (0.142−24.868)	0.633

Data are presented as n

BMI, body mass index; ASA-PS, American Society of Anesthesiologists physical status; pBT, preoperative body temperature; pWBC, preoperative white blood cell count; pPLT, preoperative platelet count; pNLR, preoperative neutrophil-to-lymphocyte ratio; pPLR, preoperative platelet-to-lymphocyte ratio; pCRP, preoperative C-reactive protein level; OR, odds ratio; CI, confidence interval

^a^Statistically significant. ^b^Logistic regression

## Discussion

We assessed the relationships between systemic inflammatory variables and postoperative complications after immediate appendectomy. An increased pNLR was identified as an independent risk factor for all postoperative complications, especially infectious complications, in patients with acute appendicitis.

Although recent accumulating evidence suggests that early surgical intervention may not always be required for acute appendicitis because of the relatively high success rate of antibiotic therapy [4–10], some studies have suggested that nonoperative management with antibiotics is not always effective in patients with acute appendicitis with a high serum CRP level, presence of an appendicolith, large appendiceal diameter, or complicated acute appendicitis [25–27]. Therefore, immediate appendectomy is considered the main treatment for acute appendicitis.

Several studies have identified various clinical variables, including older age, delayed or night operation, longer operative time, open surgery, and complicated acute appendicitis, as risk factors of postoperative complications such as wound infection, intra-abdominal abscess, and prolonged ileus [11–16]. Segev et al. [15] demonstrated that the rate of postoperative complications was higher in older adult patients with a longer delay from symptom onset to admission or from admission to surgery, complicated acute appendicitis, and a longer operative time. Regarding the optimal operation timing, some studies have suggested that delayed appendectomy may be unsafe or ineffective for adult patients with complicated acute appendicitis [11, 13–15]. In contrast, other studies have reported that delayed appendectomy is not associated with an increase in the occurrence of postoperative complications [16, 28, 29]. Laparoscopic appendectomy has become the main surgical treatment for acute appendicitis, and several studies have reported that laparoscopic appendectomy is the most effective surgical treatment for acute appendicitis and is associated with a lower incidence of postoperative complications, shorter hospital stay, and better quality-of-life scores compared with open appendectomy [30–32]. The incidence of postoperative complications after immediate appendectomy for uncomplicated acute appendicitis is generally 10–19%. However, the incidence of postoperative complications after immediate appendectomy for complicated acute appendicitis increases to 30%, and some studies have proposed that antibiotic therapy, percutaneous drainage, or interval appendectomy may have some advantages over immediate appendectomy for complicated acute appendicitis [33, 34]. In our study, the incidence of any postoperative complication was 15.5%; this is slightly higher than the incidence reported in previous studies because 48.6% of our patients were diagnosed with complicated acute appendicitis, which may affect the incidence of postoperative complications. As seen in previous studies, our results showed a significant association between postoperative complications and operative time or complicated acute appendicitis; however, we found no significant association between postoperative complications and age, operation timing, or open appendectomy.

Increased leukocyte and platelet counts are usually observed in the systemic inflammatory response. Neutrophils are the predominant leukocyte subset that is recruited to inflamed tissue by the initial innate immune system response [35], and platelets serve as a major contributor of several pro-inflammatory chemokines and possess a whole inventory of surface receptors and adhesion molecules that enable platelets to bind to leukocytes, circulating pathogens, and bacteria [36]. To our knowledge, ours is the first study to demonstrate that the pNLR is a useful predictor of postoperative infectious complications in patients with acute appendicitis. An increased pNLR indicates both a heightened neutrophil-dependent inflammatory response and a decreased lymphocyte-mediated antibacterial immune reaction, which may affect the development of postoperative infectious complications. Therefore, postoperative infectious complications in acute appendicitis patients with an increased NLR may be reduced by a combination of perioperative antimicrobial treatment (using broad-spectrum antibiotics) and anti-inflammatory therapy with immediate appendectomy, or nonsurgical treatment with antibiotics substituted for immediate appendectomy.

Our study has limitations. Because this was a retrospective study conducted in a single institution with a small sample size and patient selection bias, there were differences in patients’ characteristics, which may have led to biased estimates of postoperative complications. Moreover, it is difficult to precisely diagnose other complications with different severities. Although our findings should be interpreted with caution, we believe that their potential clinical significance justifies further investigation.

## Conclusions

Our study demonstrated that the pNLR was the most useful predictor of postoperative complications, especially infectious complications, after immediate appendectomy for acute appendicitis. Although further investigation is required to verify the utility of the pNLR as a predictor of infectious complications after immediate appendectomy for acute appendicitis, a high NLR in patients with acute appendicitis before appendectomy may contribute to the prediction or prevention of postoperative infectious complications.

## Data Availability

The datasets used and/or analyzed during the current study are available from the corresponding author upon reasonable request.

## Abbreviations

NLR: Neutrophil-to-lymphocyte ratio
PLR: Platelet-to-lymphocyte ratio
pWBC: Preoperative white blood cell count
pPLT: Preoperative platelet count
pCRP: Preoperative serum C-reactive protein level
CT: Computed tomography
C-D: Clavien-Dindo classification
ROC: Receiver operating characteristics
AUC: Area under the ROC curve
OR: Odds ratio
CI: Confidence interval
BMI: Body mass index
